# Applauding with Closed Hands: Neural Signature of Action-Sentence Compatibility Effects

**DOI:** 10.1371/journal.pone.0011751

**Published:** 2010-07-28

**Authors:** Pia Aravena, Esteban Hurtado, Rodrigo Riveros, Juan Felipe Cardona, Facundo Manes, Agustín Ibáñez

**Affiliations:** 1 Laboratory of Experimental Psychology & Neuroscience, Institute of Cognitive Neurology (INECO), Buenos Aires, Capital Federal, Argentina; 2 Laboratory of Cognitive Neuroscience, Universidad Diego Portales, Santiago, Chile; 3 Doctoral Program, Psychology School, Pontificia Universidad Católica de Chile, Santiago, Chile; 4 Institute of Neuroscience, Favaloro University, Buenos Aires, Capital Federal, Argentina; 5 National Scientific and Technical Research Council (CONICET), Buenos Aires, Capital Federal, Argentina; The University of Western Ontario, Canada

## Abstract

**Background:**

Behavioral studies have provided evidence for an action–sentence compatibility effect (ACE) that suggests a coupling of motor mechanisms and action-sentence comprehension. When both processes are concurrent, the action sentence primes the actual movement, and simultaneously, the action affects comprehension. The aim of the present study was to investigate brain markers of bidirectional impact of language comprehension and motor processes.

**Methodology/Principal Findings:**

Participants listened to sentences describing an action that involved an open hand, a closed hand, or no manual action. Each participant was asked to press a button to indicate his/her understanding of the sentence. Each participant was assigned a hand-shape, either closed or open, which had to be used to activate the button. There were two groups (depending on the assigned hand-shape) and three categories (compatible, incompatible and neutral) defined according to the compatibility between the response and the sentence. ACEs were found in both groups. Brain markers of semantic processing exhibited an N400-like component around the Cz electrode position. This component distinguishes between compatible and incompatible, with a greater negative deflection for incompatible. Motor response elicited a motor potential (MP) and a re-afferent potential (RAP), which are both enhanced in the compatible condition.

**Conclusions/Significance:**

The present findings provide the first ACE cortical measurements of semantic processing and the motor response. N400-like effects suggest that incompatibility with motor processes interferes in sentence comprehension in a semantic fashion. Modulation of motor potentials (MP and RAP) revealed a multimodal semantic facilitation of the motor response. Both results provide neural evidence of an action-sentence bidirectional relationship. Our results suggest that ACE is not an epiphenomenal post-sentence comprehension process. In contrast, motor-language integration occurring during the verb onset supports a genuine and ongoing brain motor-language interaction.

## Introduction

Bodily actions, such as gestures or emotional body language, are finely intertwined with language during natural speech. For example, when the president of a country is talking about the big steps on the road to consolidation, he/she is likely to automatically and without effort extend his/her hands in a big step gesture. But if the opposite gesture is performed (e.g., stretched the hands) during the same sentence, the overall meaning of the speech may appear incongruent. It appears that our brains couple motor and semantic processes together towards a specific significance. How does the brain produce such interactions? Moreover, do subtle aspects of movement, such as hand-shape in a limb action (e.g., open or closed) during language processing, also imply motor-language interactions in the brain? Our study looked for specific action-sentence compatibility effect (ACE) by examining motor and semantic processes indexed by Event Related Potentials (ERPs).

Given the systematic involvement of the motor system in language processing as shown by neurophysiological and behavioral studies (for reviews, see, [1], [2]), the coupling of neural networks between language understanding and action is no longer a matter of debate. However, the interpretation of this claim still confronts theoretical postures about the role of the motor system in language. More specifically, current research is still evaluating the sufficient and necessary implication of motor systems for language understanding opposed to debating their facilitation or cooperation. In addition, mutually bidirectional implications between language and motor processing are not well described.

The idea that conceptual knowledge is mapped onto sensory-motor systems (see for example [3]) comes from a series of neuropsychological studies. The groundwork of this hypothesis was the investigation of the neural dissociation of semantic knowledge for different object types. Warrington and Shallice [4] first reported that sensory attributes are salient features for the identification of animals or fruits. In turn, functional (motor) attributes are critical characteristics for the identification of tools. Another study demonstrated that this difference not only exists between categories but also within them, e.g., the specific contributions of motor channels within a modality-specific category of artifacts ([5] for reviews, see, [6], [7]). Hence, the motor repertoire becomes a semantic repertoire.

In the language understanding literature, this modality-specific activation in conceptual knowledge has been interpreted as reflecting a mental simulation, that is, an internal enactment of the sensory-motor experience during language comprehension [8]–[11]. Simulation theory has claimed that the internal enactment of motor language would engage specific areas of the motor cortex, which control the simulated effector of the action [12]–[18]. Recent reports based on ERP [19] and Magnetoencephalographic (MEG) studies [20] have confirmed that action words denoting motor programs of different effectors activate specific motor processes in the brain, and this activation occurs early after stimulus presentation. Nevertheless, some criticisms have been raised about the radical hypothesis of motor-language interaction (e.g., [21]–[24]). Specifically, it has been argued that the available empirical evidence does not support the claims of sensory-motor resonance as a causal mechanism for bringing language comprehension and human communication within the realm of the motor system.

Far from adhering to or rejecting these hypotheses, we call upon a more balanced method to consider the brain basis of language and cognition as interplay between multiple cognitive domains. Large-scale neural networks are formed dynamically, involving several parts of the cortex that are needed for one specific task [22], [25]–[28]. Meaning and comprehension appear to be general processes of cognition, and therefore, they are the bases of language, gestures, or action [29]–[40]. Thus, brain processes linked to body action should be engaged during comprehension. From this point of view, semantic content would be shared between motor processing and linguistic knowledge. When these two processes (motor and linguistic) are concurrently performed, the common neural resources should cooperate, and language should facilitate actual movement when compatible. Several studies have reported this facilitation effect with single action words [12], [16], [41]–[46]. However, word properties (including their sensory-motor attributes) are contextually dependent [47]–[48] and not static. To investigate the interplay between action-language and the motor system towards access to meaning, it is necessary to consider how the sentence context modifies the action verb.

Several behavioral studies have provided evidence for motor resonance during sentence comprehension. One particularly refined paradigm is the ACE that was first introduced by Glenberg and Kaschak [10]. In a sentence–sensibility–judgment task, subjects were presented with sentences encoding an action involving an upper limb movement either towards or away from the subject (e.g., sentence encoding movement away from the subject: *you give Liz the toy*). They controlled the movement required for the response to the sensibility-judgment task. Thus, the movement implied by the action of the sentence is either compatible or incompatible to the response action. As expected, sentence processing times were significantly faster in the compatible condition. ACE has been replicated in other experiments that have demonstrated the robustness of the effect (e.g., [49]–[57] for an overview see, [1]). Because only ACE behavioral paradigms have identified the cross-talk between motor structures and action-sentence comprehension, several questions remain about the brain correlates of this process. Thus, an exploration of the neural markers of the ACE paradigm in semantic processing and the motor response may yield important advances in the neuroscience of action-sentence blending.

Within the motor-language debate, the aim of our study was to investigate brain markers of bidirectional impact between language comprehension and motor process. It was our intention to account for a genuine and online interaction, hence we combined ACE and ERP for the first time regarding the motor-to-semantics direction in addition to the previously studied semantics-to-motor direction.

Additionally, other relevant aspects of this study are detailed here:

- Since previous studies have investigated only general aspects of motor action such as the effectors or direction of motion [10], we aim to investigate the neural markers of the motor-language relationship at a subtle aspect of the action (hand-shape).
- Most of ACE experiments have utilized first person sentences, thus they do not avoid the possibility that the activation of motor processes is due to imagery processes and therefore explain the phenomenon using confounding variables. We aim to investigate cortical responses of ACE with no first person implicated stimuli to study the robustness of effect in low imagery processes.
- We look to explore motor-language integration with independence from the intention or attention of the subjects. This could be achieved by an ACE task with motor aspects that were not relevant to the task, then it would be suggested that the compatibility effect is automatic and independent from cognitive control (e.g., attention).

The technique of ERPs is a precise tool regarding time resolution (on the order of milliseconds) that incorporates the recording of ongoing electrophysiological activity using electroencephalography (EEG). ERPs result from the synchronous activation of neural subpopulations that occur in response to events (sensory, motor or cognitive). ERPs are the sum of the activity of excitatory postsynaptic potential and inhibitory postsynaptic potential activated in response to each new stimulus.

Semantic processing has been tracked with the N400 component, a large negative deflection in the ERP occurring approximately 400 ms after the presentation of a word. Typically, the N400 is larger when a stimulus is difficult to integrate into a previous semantic context [58]. The N400 effect has been reported for semantic violations in language and for the processing of other meaningful stimuli (e.g., [29], [59]–[61]). N400 is thought to reflect the activation of amodal semantic memory [62]. In our experiment, the content of the sentences was related to the hand-shape response. Consequently, the compatible or incompatible conditions were a combination of semantic processing and motor performance (participant hand-shape response). Because one of the meaningful parts of the incompatible condition of this experiment was not linguistic (motor response), we expected to find an N400-like modulation. N400-like effects are not restricted to linguistic stimuli [63].

The movement-related cortical potentials (MRCP) associated with self-paced movements are considered a measure of motor cortex excitability [64] and allow the exploration of cortical changes related to motor preparation and execution. The first component related to this study is a negativity measured over Cz beginning shortly before the response onset (−90 ms) that has been called motor potential (MP; [65]) or late motor-related potential (late MRP; [66], [67]), which is likely to represent pyramidal neuron activity in the primary cortex (M1) at motor execution. MP amplitude modulation has been associated with the rapidness and precision of movement [65], [68] and also with short-term training effects [66]. We expected higher MP amplitudes for the compatible condition, reflecting that sentence content facilitates the precision and quickness of a compatible action. The second component consistently observed was a peak over Cz after movement onset (200–300 ms) similar to a re-afferent potential (RAP). RAP is an index of movement-related sensory feedback to the primary sensory-motor cortex [67] and is considered an indicator of attention [66]. Higher RAP peaks were expected for the compatible condition, indicating that the facilitation of bottom-up attention on task-relevant information would optimize action performance.

In the present work, we aim to introduce a novel perspective to the realm of action-language comprehension by supporting the *bidirectionality hypothesis* as an integrated method of understanding the motor-language interaction in comprehension. This hypothesis claims that action-language comprehension and motor processes share neural resources that co-operate mutually; that is to say that motor processes influences the comprehension of the action sentence, and action sentence comprehension influences the motor process. In a recent behavioral study, Kelly, Özyürek and Maris [69] demonstrated for the first time the mutual influence of gesture and speech stimuli in language comprehension. We aim to extend this bidirectional interaction hypothesis to the realm of brain markers of actual actions and language processing.

To test the bidirectionality hypothesis, we conducted an ACE study under the following prediction: if semantic content is shared by motor and linguistic processes, when both of them occur simultaneously, shared neural resources should co-operate bidirectionally. Specifically, sentence comprehension should facilitate actual movement when compatible, and at the same time, incompatible action should disrupt comprehension of the sentence.

To prove this prediction, we utilized an ERP technique that measured both stimuli (sentence) and response (action) cortical processes. What is expected particularly is: (a) if motor response is facilitated by the compatible sentence comprehension, the motor potentials (MP and RAP) should be larger in this condition because of the quickness and precision of the facilitated movements. (b) if motor processes impact the semantics of the sentence, it would be expected that during cortical semantic processing, an incompatible motor process elicits a semantic incongruence manifested by the N400-like component.

If the bidirectionality hypothesis is not fulfilled, any of the predictions (a or b) would not be present. Each prediction only accounts for evidence of a unidirectional effect. Hence, the satisfaction of only one of them is not sufficient to argue for their vice versa effect (if only (a) is accomplished, it could not be argued that motor action impact sentence comprehension; if only (b) is accomplished, it could not be argued that semantics primes the motor process effect).

Consequently, if no motor effect in the cortical processing of semantic processes is observed (no modulation of the N400-like component), the bidirectionality hypothesis fails. Also, if no semantic effect in the cortical processing of motor response is observed (no modulation of motor components, MP and RAP), the bidirectionality hypothesis fails. As a result, if both predictions fail, the bidirectionality hypothesis also fails.

To the best of our knowledge, no single study of ACE neural signatures has been reported. This study attempts to provide temporal dynamics of detailed ACE brain markers during stimuli processing and motor responses to account for both directions of analysis (semantics-to-motor and motor-to-semantics). Thus, the current work addressed three issues: (1) a description of specific semantic and motor ERPs of ACE; (2) a comparison of the semantic and motor ERPs regarding the compatible and incompatible action-sentence effects; and (3) a comparison of the behavioral and neural markers of ACE to build a more precise understanding of the action-sentence interactions.

## Materials and Methods

### Ethics Statement

All participants read and signed an informed consent in agreement with the Declaration of Helsinki before beginning the study. The ethical committee of the cognitive neuroscience laboratory approved the study.

### Participants

Twenty-six native Spanish volunteers (14 females) aged 18 to 31 (M = 22.4 years, SD = 3.7) were included in this study. All participants were undergraduate students and right-handed as defined by the Edinburgh Inventory [70], with normal auditory acuity, normal or corrected-to normal vision, and no reported history of psychiatric or neurological illness. Regarding the response hand-shape (either open [OH] or closed [CH]), two randomly balanced groups were created: one group responded to the stimuli with an open hand (open hand-shape group [OHG]) and the other with the closed hand-shape [CHG]. No differences in age [F (1, 24) = 0.005, p = 0.94], gender [X^2^ = 0.05; p = 0.82] or educational level [X^2^ = 0.03; p = 0.94] between groups were found.

### Task

**Figure 1:**
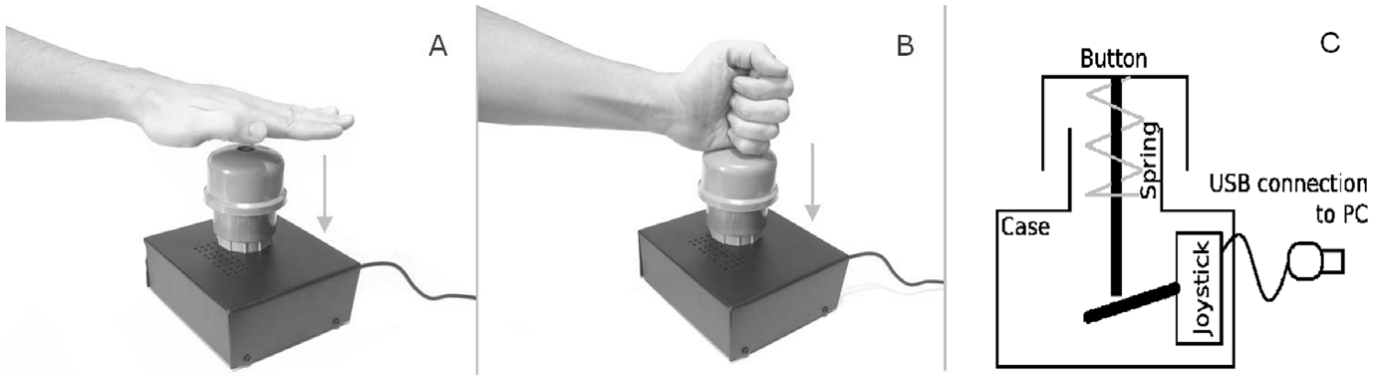
Response button and pre-assigned hand-shapes. A) OH motor response during sentence comprehension task. B) CH motor response during sentence comprehension task. C) Custom-made response button. A commonly available USB joystick with analogue sticks was adapted to detect when a participant initiated a response.

The participants listened to auditory sentences (e.g., *The show was praiseworthy, so Rocio applauded*, see Table 1 for more examples) and indicated as quickly as possible once they understood each sentence. They made this judgment by pressing a button and, using a pre-assigned hand-shape, either a CH vertical to the button or an OH (see Figure 1.A and B). Similar CH and OH responses have been reported previously to be modulated by sentence content [50].

**Table 1:** Example of stimuli used in the experiment and their approximate English translation.

Category	Sentence	English Approximate Translation
OHS	*El espectáculo era digno de alabanza, Rocío aplaudió.*	*The show was praiseworthy, so Rocio applauded*
CHS	*Tenía que clavar el clavo muy derecho, José lo martilló*	*He needed to drive the nail correctly, so Joseph hammered it.*
NS	*Hace tiempo que quería ver a su abuela, Amaro la visitó*	*After waiting a long time to see his grandmother, Amaro visited her*

The sentences were categorized according the hand-shape of the action encoded in OH sentences (OHS), CH sentences (CHS) and neutral sentences (NS). Final target verbs are underlined.

### Stimuli

One hundred fifty-six sentences in Spanish, previously validated (see below), were considered as stimuli (see Stimuli S1). One hundred and four sentences encoded hand actions; half contained verbs encoding an action with an OH and half encoded a CH. In addition, 52 neutral sentences encoding no action or an action different to a hand action were included. Sentences were categorized as OH sentences (OHS), CH sentences (CHS) and neutral sentences (NS) (Table 1).

All sentences were third-person whose critical verb was in undefined preterit tense (English's Simple Past Tense) and was always placed as the last word of the sentence. Relevant linguistic variables were matched between lists (such as Transitivity, Situation Aspect, and content of the clauses; see Methods S1).

Also, the number of syllables of the final target word (M = 2.46 syllables, SD = 0.08 in OHS; M = 2.53 syllables, SD = 0.08 in CHS; and M = 2.53 syllables, SD = 0.08 in NS; F(2, 153) = 0.25, p = 0.77), as well as the frequency of use (moderate levels) for those targets, was controlled. The overall length in time of sentences was 4.57 s (SD = 0.06 s). Audio files of each trial were edited with 400 ms of silence at the beginning and 200 ms at the end. The onset of the target verb within the sentence was 4.05 s (SD = 0.06; 2.92 s minimum, 5.64 s maximum).

### Stimuli validation

A rating study was designed to validate stimuli (see Methods S1). Lists of CHS, OHS and NS were controlled for sentence length, final word length and frequency (moderate). In addition, predictability, prototypicality and the degree of manual specificity of hand sentences were controlled. Predictability was defined as how easily the final verb was to determine from the previous sentential context. Prototypicality was defined as how representative of the pertinent hand-shape (CH or OH) was the manual action encoded by the sentence. The degree of manual specificity was related to manual aperture or closure. In other words, the degree of manual specificity defined how open or closed the hand-shape must be to perform the determined action encoded by the sentence.

Only highly predictable sentence endings were considered. NS presented statistically enhanced levels of predictability compared with OHS and CHS, but no difference between OHS and CHS was observed. All hand actions encoded by sentences (OHS and CHS) were highly prototypical of their shape. However, CHS was reported as more difficult to perform with the incompatible movement (OH) compared to OHS. Consequently, we expected more accentuated ACE motor responses in the CHG. Higher prototypicality implies an enhancement of facilitation for the compatible movement because the information about the hand-shape of movement is strongly reinforced by the very specific hand-shape content of the sentence. The detailed process of validation can be found in Methods S1.

### Measurement of OH and CH responses

Responses were captured using a custom-made response button (Figure 1) that could be depressed 4 cm and was mounted on a spring sufficiently stiff to hold the button and hand in the normal position. The button size allowed the hand to rest comfortably in either a closed or an open position. A USB joystick with analog sticks was mechanically adapted (see Figure 1.C) so that one axis of one stick would measure how deep the button was depressed to detect when a participant initiated a response. This design allowed a relatively long range of the button that emphasized the hand action characteristic of the response.

At 8 bits of resolution per axis, the stick measured values between −128 and 127 with 0 being the center and −128 being fully depressed (half the range of the stick was unused). When a measurement was more negative than some pre-established threshold (to rule out spurious responses due to measurement noise), a response was detected. In this way, both short and complete depressions could be detected, despite the long run of the button.

### Procedure

The experiment was conducted in an electrically- and sound-shielded room under dim lighting conditions. The subjects were comfortably seated behind a desk facing a computer display. The button was located on the right side of the desk so that participants could easily press it. Participants were instructed to listen to the sentences and indicate by button press as quickly as possible the moment of comprehension. They were asked to keep both hands in the pre-assigned hand-shape throughout the experiment (see part A and B of Figure 1); the right hand was placed over the button and the left hand over the desk. Also, to ensure that participants were attending to the stimuli, they were told that they would be asked about the content of the last word of each sentence at the end of the experiment. Subjects had a training session (5 trials) for familiarization them with the task.

Each trial began with an ocular fixation cross at the center of the monitor that appeared 300 ms before the beginning of the sentence and disappeared 800 ms after the response. The inter-stimulus interval was set at 150 ms. Sentences were recorded by a female native Spanish speaker. Auditory stimuli were recorded and set using Audacity 1.2.6 software. Experimental presentation and data collection were performed with Python software. The 156 trials were uniformly distributed over the three conditions of sentences in a counterbalanced list, ensuring that the same condition was not appearing more than twice consecutively (see Table 2).

**Table 2:** Conditions of the experiment.

	OHS	CHS	NS
OHG	Compatible condition	Incompatible condition	Neutral condition
CHG	Incompatible condition	Compatible condition	Neutral condition

Interaction of the type of response (OHG or CHG) and content of the sentences (OHS, CHS, NS) in three categories (compatible, incompatible and neutral).

### ERP Data Acquisition and Processing

Electroencephalographic (EEG) data were acquired with a 128 channel Electrical Geodesics Inc. (EGI) system, GES300, consisting of Hydrocel Geodesic Sensor Net, Net Amps and Net Station software (Electrical Geodesics Inc.). EEG data were sampled at 500 Hz and 0.1–100 Hz analog filtered. Impedances were kept under 50 kΩ. EEG data were continuously recorded by default to vertex and offline re-referenced to linked mastoids (motor responses) and average electrodes (N400-like component). A band pass digital filter between 0.5 and 30 Hz was applied to remove unwanted frequency components. EEG data were segmented offline into 1 s epochs spanning from 200 ms pre-stimulus to 800 ms post-stimulus for stimulus-locked segments and 1500 ms epochs from −500 ms to 1000 ms for hand response-locked segments. EEG channels with visually detectable artifacts (e.g., eye blink, channel drift and gross movement) were isolated using the Net Station Waveform Tools (NSWT) and discarded from the analysis. In addition, automatic ICA and adaptive autoregressive modeling was performed to discard further artifacts.

### Data analysis

Reaction times (RTs) were calculated for each subject in each condition (compatible, incompatible and neutral). Outliers with RTs outside +2.5 SD were deleted.

For ERPs, a strategy for channel location reported previously was used (e.g., [71]–[72]). A time-course analysis for 9 representative electrodes (11(Fz), 24(F3), 36(C3), 52(P3), 62(Pz), 92(P4), 104(C4), 124(F4) and 129(Cz)) in the compatible, incompatible and neutral condition for OHG and CHG was implemented. After an electrode×category analysis of all ERPs (see Methods S2), the vertex site was selected based on bigger amplitudes and differences between categories. The Cz region has been reported previously as the main site for N400 [73] and motor responses [66]. Although ERP figures show single electrodes, a region of interest (ROI) of those 6 electrodes around the maxima of MRCP and N400-like (vertex) effects were chosen to analyze the ERPs. This analysis is consistent with previous reports for maxima location of motor responses [66] and the N400-like component [74]. ERP figures shows those selected channel locations. ERPs were analyzed by considering mean amplitude values. The MRCP consisted of two components (similar to those reported in [66]), an MP peaking between −90 and 50 ms immediately following the onset of the movement followed by a clear positive deflection, resembling the RAP, peaking from 200 to 300 ms after movement onset was considered. Both MRCPs were triggered by the zero time response automatically detected by the computer when the button was activated. The N400-like component was analyzed 350–650 ms after stimulus onset (final target word). Because of the observed early differences at 0–150 ms and 150–300 ms windows, we performed a preliminary analysis over those windows. Those comparisons yielded no statistical effect. Consequently, we did not include them in this report. By including the N400 and the MRCPs triggered by semantic stimuli and motor response respectively, we were able to measure the possible bidirectional effects on action and language processing.

### Statistical analysis

Repeated measures of Analysis of Variance (ANOVA), with group as the between-subject factor (OHG and CHG) and category as the within-subject factor (compatible, incompatible and neutral), were performed for behavioral and ERP measures. For example, CHS in the OHG and OHS in the CHG were considered incompatible categories (see Table 2). An additional factor, stimulus content (NS, OHS and CHS), was introduced when necessary. Matlab software was used for offline processing and analysis of ERP data. ANOVA degrees of freedom were corrected using the Greenhouse-Geisser method to adjust the unvaried output of the repeated measures ANOVA for violations of the compound symmetry assumption. When the covariances are not equal and all the variances are not equal, this method adjusts the degrees of freedom in the ANOVA test in order to produce a more accurate significance (*p*) value. Tukey's HSD method was used for the calculation of post-hoc contrasts.

## Results

### Behavioral measures

#### Content effects

Regardless of ACE, an effect of stimulus content was significant (F(2, 48) = 8.07; p<0.001). Post hoc comparisons (MS = 776.34; df = 48.00) showed that NS elicited a shorter response (M = 611 ms, SD = 50) compared with OHS (M = 920 ms, SD = 70; p<0.001) and CHS (M = 793, SD = 60; trend: p = 0.58). No differences between OHS and CHS were obtained (p = 0.23). A group effect elicited a trend (F (1, 24) = 3.48, p = 0.07), suggesting a faster processing of the CHG (M = 698 ms, SD = 58) compared to OHG (M = 852 ms, SD = 58).

#### ACE

**Figure 2:**
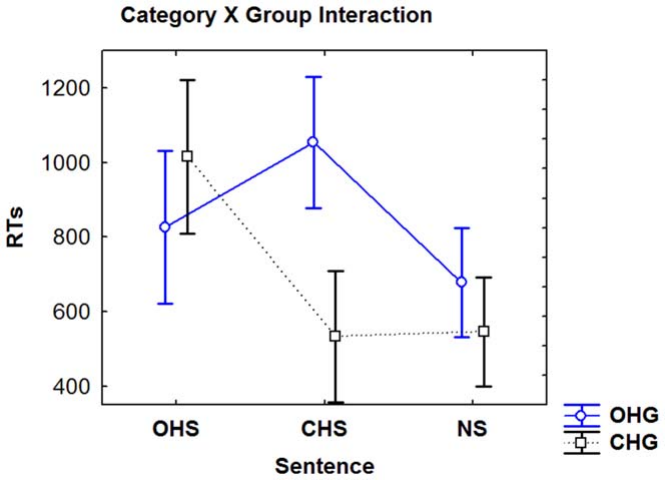
RTs of ACE for OHG and CHG. In the CHG, the compatible sequence trials comprised CHS and the incompatible comprised OHS. In the OHG, the compatible and incompatible effects were opposite. Vertical bars denote 0.95 confidence intervals.

A strong ACE of category was found (F (2, 48) = 8.07, p<0.001). The incompatible category presented longer RTs (M = 1034 ms, SD = 102) compared with the compatible (M = 679 ms, SD = 92) and neutral categories (M = 611 ms, SD = 70). Regarding groups results (Figure 2), a group×category interaction was significant (F (2, 48) = 10.57, p<0.001). Both groups seemed to elicit an ACE, although this was more accentuated in the CHG. Post hoc comparisons performed over this last interaction (MS = 96.21, df = 67.00), showed that in the CHG, incompatible responses (M = 1015 ms, SD = 99) elicited longer RTs compared with compatible (M = 533 ms, SD = 85; p<0.001) and neutral stimuli (M = 545 ms, SD = 70; p<0.005). No differences between compatible and neutral stimuli were observed (p = 0.99). In the OHG, responses from compatible stimuli (M = 826 ms, SD = 99) were shorter than responses from incompatible stimuli (M = 1053 ms, SD = 85; p<0.05). Neutral stimuli (M = 677 ms; SD = 70) elicited shorter responses than incompatible ones (p<0.05).

In brief, behavioral effects regarding stimulus content suggest that more predictable sentences (NS) elicit shorter responses compared with OHS and CHS. The CHG presented a trend towards a more rapid response. More importantly, ACEs was present in both groups, although it was accentuated in the CHG.

### ERPs analysis

#### Effects related to stimulus (N400-like)

**Figure 3:**
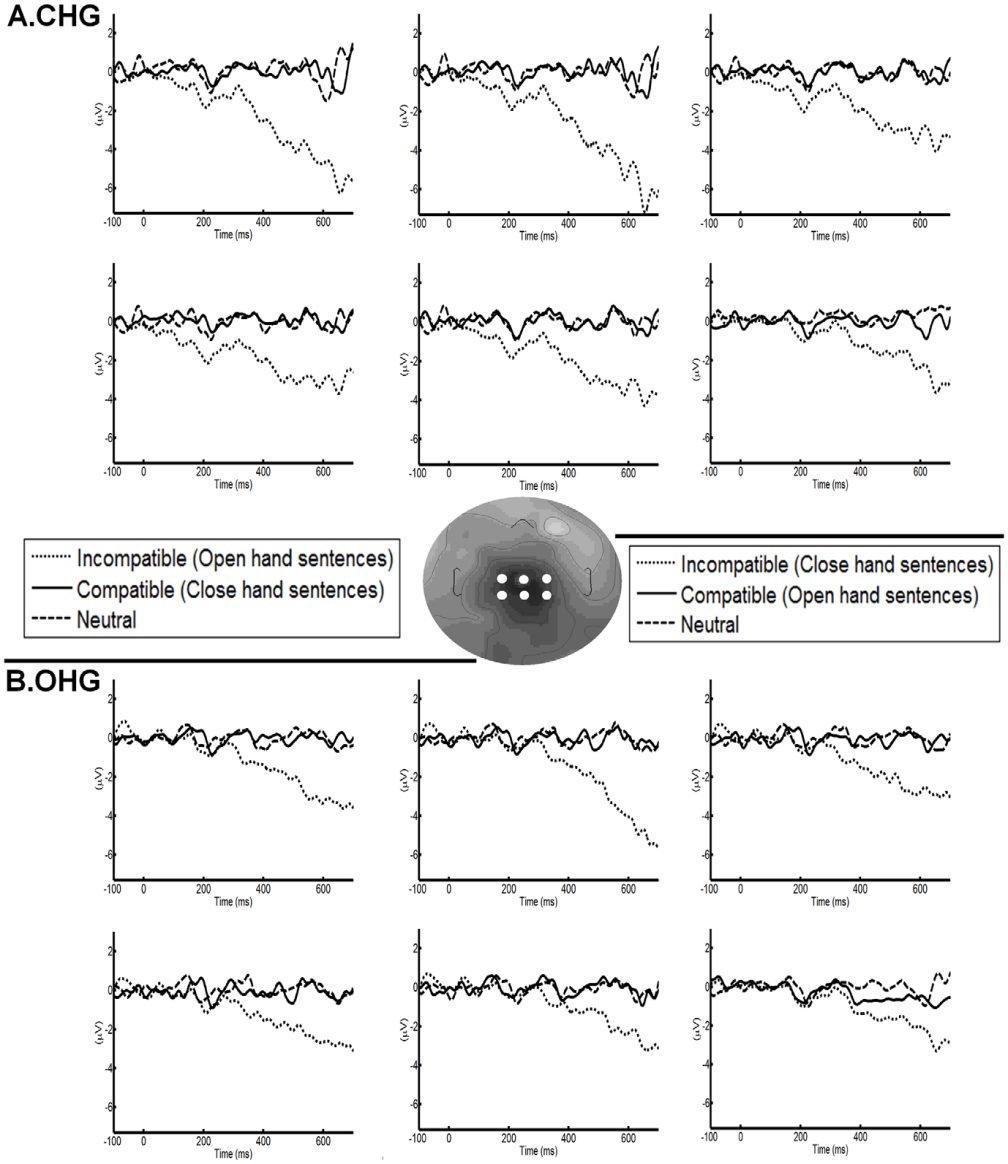
N400-like effect for OHG and CHG. The channel locations of selected electrodes are shown in the grey circle. Note that the incompatible stimuli which elicits N400 amplitude enhancement are OHS in CHG (Figure 3.A) and CHS in the OHG (Figure 3.B).

As illustrated in Figure 3, incompatible stimuli exhibited an N400-like component around Cz in both OHG and CHG. An ANOVA with category as the within-subject factor and group as the between-subject factor yielded an effect of category (F (2, 48) = 65.27, p<0.001). The incompatible category (M = −3.04 µV, SD = 0.33) presented more negative values compared with the compatible (M = 0.06 µV, SD = 0.38) and neutral categories (M = 0.01 µV, SD = 0.14). Post hoc comparisons showed statistical differences between compatible and incompatible categories (p<0.001), as well as between neutral and incompatible categories (p<0.001). However, no differences between neutral and compatible categories were observed (p = 0.99). Neither an effect of group (F (1, 24) = 0.79; p = 0.38) nor a group×category interaction effect was found (F (2, 48) = 0.84; p = 0.43).

To evaluate whether stimulus content affected the N400-like component, an ANOVA of stimulus content × group was performed. As stated in Table S1, sentences with hand content (OHS and CHS) elicited enhanced N400-like amplitudes compared with NS, but this effect was affected by the type of hand-shape response (content×group interaction: (F(2, 48) = 48.31, p<0.001; see post hoc effects in Table S1). Because those NS were rated more predictable, the effect can be explained by predictability, as one robust modulator of the N400 component [75].

The overall results of N400-like amplitude suggest an ACE which discriminated incompatible stimuli from compatible and neutral stimuli. In addition, this component seemed to be affected by the predictability of the sentences, because OHS and CHS elicited more negative amplitudes than NS. No group differences were found in this component.

#### Effects related to response: Motor potential (−90 to 50 ms)

**Figure 4:**
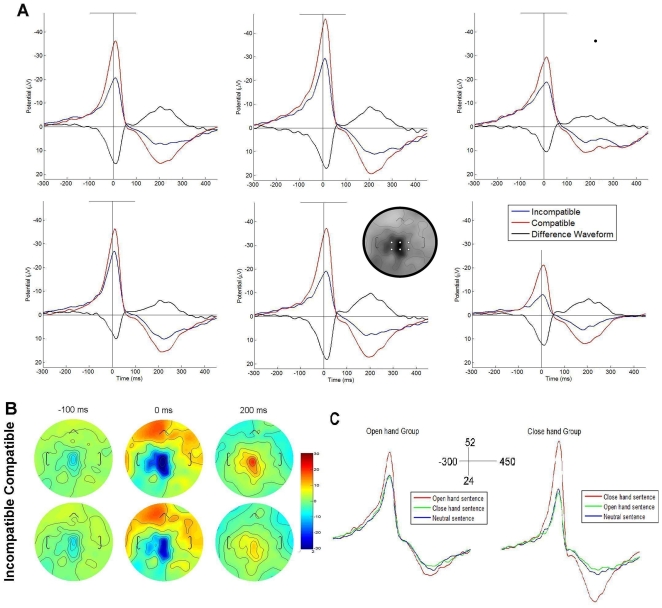
ERPs from motor responses. A) Compatibility effects and difference waveforms for MP and RAP. B) Voltage maps from compatible and incompatible categories in the −100, 0 and 200 ms. C) Selected electrodes (Cz) showing compatibility effects in OHG and CHG. Channel locations are shown in the grey circle inside the figure.

In the early time-window related to the response, a category effect was observed (F (2,48) = 32.85; p<0.001). Conversely to the N400-like components, post hoc effects (MS = 28.01, df = 48) indicated an enhanced amplitude for the compatible category (M = −21.39 µV, SD = 1.75; p<0.001), compared with the incompatible (M = −11.14 µV, SD = 1.36; p<0.001) and neutral categories (M = −11.03 µV, SD = 0.91). No differences were observed between these categories (p = 0.99). Figure 4.A shows the compatibility effects and the difference waveforms and Figure 4.B illustrates the voltages maps.

In addition, a category × group interaction was obtained (F (2, 48) = 8.21, p<0.001). Although both groups presented the same pattern, with the compatible category eliciting a greater amplitude, this effect was more pronounced in the CHG (see Figure 4.C). Post hoc comparisons performed over this last interaction confirmed that the compatibility effects were larger in the CHG (see Table S2). In this group, the post hoc effects of compatibility yielded statistical significance.

In brief, the early ERPs elicited by the motor response were modulated by compatibility effects, suggesting action-sentence compatibility facilitation. Moreover, these effects were accentuated in the CHG.

#### Effects related to response: Late motor response RAP (200–300 ms)

The analysis of late motor effects yielded similar results to MP; compatibility modulation was observed (F (2, 48) = 44.56, p<0.001). Post hoc comparisons (MS = 12.11, df = 48) of this effect indicated that compatible stimuli elicited enhanced positivity (M = 13.79 µV, SD = 1.007) compared with incompatible (M = 5.92 µV, SD = 0.86; p<0.001) and neutral stimuli (M = 5.88 µV, SD = 0.56; p<0.001). A group×category interaction effect was also found (F (2, 48) = 12.7; p<0.001). Post hoc comparisons (MS = 13.47, df = 70.54) revealed similar effects of CHG and OHG as the early motor response (see Table S3).

In conclusion, RAP seemed to be affected by compatibility, suggesting an enhanced motor facilitation in response to compatible stimuli. Nevertheless, when group comparisons were considered, this effect was only statistically significant in the CHG.

## Discussion

The present study sought to reveal an interaction between action sentence comprehension and motor processes by analyzing the impact of each on RTs and neurophysiological correlates of the semantic process and action performance. The present work was performed with the aim to test the bidirectionality hypothesis and results confirmed the needed predictions to fulfill it.

Behaviorally, the effects of action-sentence compatibility were consistent with previous findings [10], [50], [51], [54], [76]–[78]. In this design, the ACE was based on the interaction between response hand-shape (CH or OH) and hand-shape actions encoded in the sentence. As expected, subjects were quicker to press the response button when the hand-shape required to respond was compatible with the hand-shape implied by the sentence. Similarly, RTs were significantly slower on the incompatible action sentences. This can be interpreted as evidence that action-language comprehension has a multimodal priming effect on action performance and to this level of specific motor attribute of movements (hand-shape) and with third-person sentences (see also [50]). As addressed previously, behavioral data indicated that the NS elicited a shorter response than both manual sentences. The best explanation of the rapidity of response in the neutral condition might be the facilitation by predictability effect.

ERPs analyses, an excellent tool to investigate the temporal and functional mechanisms supporting language comprehension, refined the behavioral understanding of the ACE. In this study, N400 from semantic processing was time-locked to the target final verb, and the motor MRCPs were time-locked to the onset of the button activation. Our results revealed that in the semantics-to-motor direction: (a) cortical markers of motor process (MP and RAP) are affected by semantic effects, and in the motor-to-semantics direction; and (b) brain markers of comprehension processes (N400-like) are modulated by motor effects. Thus, both cortical processes of the task show modulation by their counterpart. Basically, language and action, both co-operators of the coupling, impact each other. Therefore, the bidirectionality hypothesis was supported.

### Overall results

The shorter latency of reaction times for the compatible condition is indicative of the action that was effectively facilitated by the sentence, inducing a quicker and more precise movement. This was confirmed cortically as larger amplitudes of MP associated with central motor speed and the control of force [65], [68] that occurred with the action-sentence compatibility. As addressed below, this facilitation effect also had a corresponding cortical effect with a larger RAP on action-sentence match, suggesting an enhancement of bottom-up attentional resources that in turn facilitated quickness and response precision.

Regarding the relationship between the behavioral data and ERP in the neutral condition, we found an N400-like effect, similar to that in the compatible condition and shorter RTs for NS. NS presented no difficulties for semantic integration of action because no neural substrates are shared between the sentence and action in a compatible/incompatible manner in this condition. Moreover, as detailed in the Materials and Methods section, the NS list involved more predictable sentence endings than the other two lists. Consequently, this should explain the shorter RTs for NS. The predictability modulated the RTs and N400 because predictability facilitates the expectation of semantic integration [75], [79]–[82]. Then, both predictability and compatibility are different effects of facilitation that account for the balance between conditions.

### Semantic Effects

The negativity elicited by incompatible stimuli in both OHG and CHG resembles the N400 component usually observed for semantic mismatches [58], [83]. Thus, when subjects prepared the action (e.g., press a button with CH), the presentation of a sentence whose hand-shape content was incompatible (e.g., *The show was praiseworthy, so Rocio applauded*) elicited a larger N400-like component compared with the compatible sentence (CH in e.g., *Her teeth were dirty, Mary brushed them*). This component could account for cross-talk between action-sentence comprehension resources and the activation of motor systems. The semantic process is related not only to linguistic stimuli but also to the motor properties of the task and generated a multidimensional activation that could have influenced processing soon after the appearance of the final verb. Similar N400-like effects not restricted to linguistic stimuli have been reported ([29], [61], [84] for a review see [63]). Thus, N400-like effects suggest a semantic multimodal integration of meaningful stimuli.

The observed flatness of and to some extent noisy N400-like waveforms can be explained by the combination of several factors: (a) the lack of a discrete onset of auditory stimuli (e.g., [85]–[88]); (b) dynamic and not-static onset triggered by hand movement (similar non-static onset has been reported: [29], [31], [89]–[91]); and (c) enhancement of motor noise induced by a strong motor preparation (hand response positions). In brief, our N400 waveforms can be explained by similar morphology reported in paradigms using auditory and non-static onset stimuli as well as by an increased motor preparation of hand responses.

Despite the early observed differences (occurring before N400-like time window), no ACE was presented at 0–150 and 150–300 ms windows (see Material and Methods section). N400-like paradigms using auditory stimuli (e.g., [85], [87], [88]) or non-static onsets triggers (e.g. [29], [31], [89]) usually exhibit increased noise and small random effects at early windows explained by the continuous (non-discrete) stimulus presentation format. Nevertheless, even if this difference had been significant, that would not be inconsistent with our results, since the early effects would be explained by a postural priming of motor preparation for the movement. Since the participants were asked to keep, throughout the experiment, both hands in the pre-assigned hand-shapes, the motor preparation was compatible or incompatible with stimuli throughout the experiment. Previous reports have shown that posture modulates behavioral and cortical semantic processing [51], [92]–[94]. N400 is a robust marker of conceptual integration, and it has been demonstrated that when some clues can be anticipated, there are early effects (spoken words: [85]; video clips: [29], [31], [89]). Consistent with those previous results, the early deviation occurring before the N400 window (although non-significant) observed in the present study can be explained by automatic contextual anticipation of compatible/incompatible preparatory motor activation. The hand posture for responding operates as a context to anticipate the hand posture of the sentence. This effect is even more plausible considering that the predictability of all sentences included in this study was high. Therefore, early effects would be explained by anticipatory effects of compatibility between motor preparation and sentence content. Nevertheless, present data show only significant effects near response onset and not previously at the postural stage. In sum, early observed differences between categories, although not significant, can be explained by an anticipatory effect (compatibility of semantic clues anticipated by posture) or by random noisy signals due to the above described features of our paradigm.

### Motor effects

We reported larger amplitudes of MP in the compatible condition. It has been demonstrated that a close relationship exists between the MP and central motor output (rate of force, precision of movement and speed [65], [68]). Larger MP peak amplitudes indicated that the activity of the motor cortex of subjects in the compatible condition was activated through the motor task that was performed more quickly and precisely. This could suggest that semantic priming facilitates compatible actions.

Moreover, this action-sentence effect was modulated by a specific feature of the movement, hand-shape. The difference between conditions was not related to a general aspect of motor action (e.g., effectors or direction of motion previously reported [10]). Rather, it was related to a very subtle aspect of manual action (hand-shape) and is demonstrated by the degree of precision indicated by the MP.

The RAP also increased in the compatible condition. The increment of afferent input is indicative of greater attention and proprioceptive feedback [95], [96]. During limb movement, somatosensory information is necessary for the precise adjustment of muscular innervations [97]. The semantic priming in this case involved multimodal bottom-up attention. When the referential content of the motor program was compatible with the content of the sentence, preparatory states focused resources selectively on task-relevant information to enhance behavioral performance. Thus, the facilitation effect of semantic information may induce expectations that guide attention to the semantics of an upcoming action. Increasing the attentional aspects of a movement task increases the magnitude of the RAP [98].

### Group Effects

We found lower RTs in compatible conditions and a trend for mean responses in the CHG in comparison with the OHG. This might be explained by a force effect. Muscularly, CH involves the contraction of forearm flexors that is similar to the grasping action, implying more muscle strength than OH [99]. The contraction of the muscles is related to the preparatory activity that normally increases the force of the movement and therefore the response velocity.

Greater amplitudes of MP and RAP in CHG were found. As detailed in the Materials and Methods section, CHS encoded more prototypical action regarding hand-shape. Higher prototypicality in the compatible condition involved an intensification of the facilitation effect, due to the multimodal reinforcement of the same information concerning the hand-shape of the movement. Although in the less prototypical sentences (OHS) the effect of facilitation was also present in the compatible condition (performed by OHG), this effect was minor compared with the action compatibility of the more prototypical stimuli. Thus, CHS response was easier to perform with a compatible hand movement (CH) than OHS response performed with a compatible movement (OH), resulting from the prototypicality difference. On the other hand, the force of contraction has been associated with MP amplitudes [100]–[101]. Thus, higher amplitude values of MP in CHG might be caused by the enhanced force implicated by more muscle contraction and precision.

### The novel contributions of this experiment

Our investigation is the first to provide ACE electrophysiological correlates. The ACE paradigm was designed to support the claim that the motor system supports language comprehension [10]. Nevertheless, the behavioral results of ACE only account for evidence of a priming effect of semantic knowledge in motor action. Hence, this finding does not provide sufficient reason to argue for the vice versa effect (motor action subserving sentence comprehension).

Glenberg et al. [76] found greater modulation of activity in the hand muscles while reading sentences describing the transfer of objects with the hand (*You give the pizza to Andy*). This report suggested that ongoing language processing activates the motor system. Thus, it has been proposed that sentence-action relation is not only unidirectional, but there is motor facilitation by the sentence as well as an impact on sentence comprehension by motor activation. Nevertheless, activation of the motor system does not fully account for the motor impact on language comprehension. The present findings provide a cortical correlate of action semantics in the form of an N400-like effect. Our results suggest that the incompatibility of the motor process affects sentence comprehension in a semantic way. Thus, multidimensional activation may influence processing that occurs during the appearance of a critical word of the sentence. Consequently, this study adds evidence regarding brain markers of semantic processing to previously reported ACE.

While language-induced cortical motor activity in subjects has been shown to affect motor behavior (e.g., [10], [12], [46], [50]), the present study also shows that motor activity contributes to action-sentence understanding. The N400-like effect suggests semantic interference of the action encoded by the sentence if the motor action is incompatible. Specific semantic processing is impacted by the real hand-shape. Thus, we focused on the motor task as well as language comprehension to provide evidence for sentence and motor integration. Both processes appear to mutually reinforce each other, sharpening both semantic and motor processes in a real-time multimodal blending.

Although the neural markers of interference facilitation of the motor action effects with single action words has already been studied, we report cortical correlates for the extent of the effect from the lexical level to the sentence level. We assume the extent because understanding the specificity of the action described by the lexical level of the verb (e.g. *show*) requires online semantic integration across the sentence (*The gypsy was going to read her hand, so Josefa showed it to her*). Thus, to determine the specific properties and to elicit the appropriate hand-shape of the verb *to show*, it is necessary to integrate it into the sentence context.

Another particular contribution of our study is that, because most of the investigation on ACE evaluated more general aspects of motor actions such as effector or direction of movement (but see [41]), we manipulated a finer aspect of action (hand-shape) at the behavioral and cortical level.

In contrast with most of the previous ACE experiments, we only used sentences that referred to a third person. This avoided the possibility that the motor-language interactions could be explained by mental imagery. To explore the role of the motor system in sentence comprehension, it is important to avoid the possibility that this activity came from the first person sentence content-induced mental imagery. If the motor cortical process activated by the sentence is a result of imagery, its functional role in comprehension appears to be more ambiguous [102]. The third person sentences in the present study have attenuated the possibility of imagery effects on motor activation and avoided the first person perspective that has been shown to be a critical component in the mental imagery of actions [103].

Our results suggest ongoing (and not post-sentential) brain measures of action-sentence activation of motor and language processes (see [76]). Because the RTs are measured only after the sentence is fully presented in the ACE behavioral paradigms, those studies cannot discount the possibility of an epiphenomenal post-comprehension process such as action preparation (instead of a real process of motor-language integration). Our results show that action-sentence integration implies an ongoing, not post-sentence, motor-language integration (occurring during the of verb onset) through the early stage of motor response (MP). These results support that ACE implies a genuine and ongoing brain motor-language interaction.

As we have mentioned above, in the field of motor-language research some criticisms have been raised about the radical hypothesis of motor-language interaction. We propose a perspective that can account for the coordination of processes and also consider the criticisms of the radical embodiment of language. Most of the criticisms of the radical hypothesis of the motor-language theory are parts of the general caveats of the mirror neuron system [21], [22], [24], [104], [105]. However, difficulties of the mirror neuron theory itself [106]–[108] do not necessarily apply to motor-language interactions. Under the idea that the brain uses several sources of information in a qualitatively similar manner to arrive at full comprehension, some positions support language-motor interaction and still discuss the radical mirror neuron theory [24], [109], [110]. Those positions state that empirical observation supports the notion that human communication relies on cognitive processes operating on semantic knowledge, *in addition* to motor couplings [22]. This indicates the inadequacy of a *causal hypothesis* of motor systems as the unique neural basis for language [109], [111], [112]. Along these lines, a raised criticism asserts that empirical evidence does not support the concept that language requires necessarily/causally the motor system and that neuropsychiatric research in motor pathologies contradicts the idea that language is supported only by virtue of sensorimotor processes. In line with those criticisms, our data does not support directly the radical claims of the motor-language theory. First, the co-operation between motor and semantic processes reported by our study does not contradict that these are partially dissociable processes [113]. Neural network co-operation implies a dynamic interaction of motor-language systems instead of a motor causal basement of language. This bidirectional co-operation does not support by itself the causal role of the motor system to achieve language comprehension; rather it shows a very robust interaction between processes. Radical criticisms that dispute the robustness of interaction between motor and semantic processes in language comprehension are scarce [21]. Even in the “disembodied” hypothesis [21], the “representation” of an action word have an “interface” with motor systems.

Dissociations between action and action-language have been documented in neuropsychiatric research and interpreted as a counter-argument for the embodied language theory. Effectively, there are studies that show that language is not impaired with disrupted motor regions and vice versa; apraxic patients are often but not always aphasic [114]–[116]. Nevertheless, evidence from lesions and pathologies are not univocal regarding motor-language systems [117]. For example, studies with lesions as causal effects of deficits in language comprehension show that those impairments in action-language are not explained by another cognitive impairment. Motoneuron diseases involve more affected processing of verbs/actions than nouns/objects [118], [119], frontotemporal dementia shows a similar pattern [120], [121], Parkinson's disease patients have deficits producing verbs [122], [123], and verb-processing deficits has been reported in Amyotrophic Lateral Sclerosis [124]. All of those studies have been taken as evidence that processing lexico-semantic information about action words requires the integrity of the motor system.

This inconsistent data can be understood in terms of a dynamic co-operation model, because it assumes that linguistic activity operates in the context of a large and dynamical cerebral network and the contributions of each system are shown to change over time, reflecting changes in experience [125]–[127]. For example, plasticity in compensatory organization in patients who recover from aphasia [128], [129] suggests that in neuropsychiatric syndromes, cognitive systems are partially dissociable by learning effects. The coordination view is compatible with those contradictory findings, since it does not assume a necessary and sufficient motor involvement in language but a dynamic coordination in interaction with diverse brain regions and cognitive systems.

### Limitations

The necessity of the contribution of the motor process for sentence comprehension remains to be established. Evidence for the effects of the motor system on comprehension is supported by present research but is not a sufficient argument for the radical embodiment of language. Rather, these results only provide evidence of multiple brain areas that are systematically involved in understanding, suggesting a blending of motor-semantic interactions. Studies of causal implication of the motor system in ACE are required as well as more specific semantic effects in the motor system.

Future work would assess ACE in semantic and motor pathologies in order to highlight more precise and detailed effects of motor-language network impairments. For example, it could be expected that patients with Early Parkinson's Disease (ePD, a disease that mainly affects subcortical motor structures) and also patients with early Amyotrophic Lateral Sclerosis (eALS, a disease affecting both upper and lower motor neurons in the motor cortex of the brain) would present remarkable deficits in action-sentence compatibility tasks. Moreover, direct comparisons between both pathologies would allow the exploration of the relevance of different areas in action-language comprehension. It can be expected that deficits in eALS probably would be larger than those in ePD, suggesting in that case that cortical areas are more relevant than motor subcortical areas for motor-language interaction.

The complete balance between categories regarding prototypicality was problematic in the current design. During the sentence construction process, we found that many verbs with CH carried hand-shape specificity more inherently in their semantics without the contextual restriction (e.g., the verb *to wield* or *to hammer* does not require the direct object to comprehend the CH of the action). In contrast, we found that many actions that required OH implied verbs with intrinsic semantic that promoted less accentuation of the specific hand-shape. Thus, contextual restriction must be higher to denote OH than to denote CH: by itself, a verb such as *to show* does not necessarily imply OH. One can *show an object in the house* (sentence with no specific hand-shape involved) and *show the gypsy your hand so she can read it* (sentence with OH involved). In both groups and within the two categories, the ACE was found and therefore, language-induced motor effects were likely modulated by the whole sentence (when information was integrated). However, weather the compatibility effect was incremented by the lexical level of the critical verbs or from the complete sentence event remains unclear. Future work will investigate differences between action-sentence context and verb-inherent semantics regarding the modulation of language-induced motor effects.

Another restriction of this study is that it does not directly show that motor activation is evoked during “normal” language comprehension without an action needing to be performed. Responses were required to obtain motor ERPs in our design. Nevertheless, further designs without explicit responses could compare the current results of peripheral activity in hand muscle activation (motor) and an N400-like component (semantic).

Another possible caveat is that in the present design, we were not able to determine the early stage of MRP known as Bereitschaftspotential. Nevertheless, the inter-individual variability for this component is extreme to the point that it may be absent in a number of participants [100], [130], [131]. Also, because our paradigm did not have the 2 s inter-stimuli windows needed for the early Bereitschaftspotential [100], it was not possible to observe this component. Finally, to elicit this component in the classic form, participants are required to move irregularly, not repetitively and not too fast [100], features absent in this design. Future experiments can be designed for the study of Bereitschaftspotential that can address the possible ACE modulation of this component.

### Conclusion

Within the motor-language debate, our study is the first to report brain markers of bidirectional impact between language comprehension and motor process. We account for a genuine and online interaction by combining ACE and ERP regarding the motor-to-semantics direction in addition to the previously studied semantics-to-motor direction.

Since previous studies have investigated only general aspects of motor action such as the effectors or direction of motion [10], we report neural markers of the motor-language relation at a subtle aspect of the action (hand-shape). Our results showed the robustness of ACE in low imagery processes. Motor aspects were not relevant to the task, therefore the compatibility effect appeared to be automatic and independent from cognitive control (e.g., attention).

An N400-like effect was found, indicating that the incongruence of the motor process interferes with sentence comprehension in a semantic fashion. Larger amplitudes of MP and RAP in the compatible condition suggest that semantic priming facilitates motor performance. Thus, our results evidenced, by comparing sentence processing and motor response ERPs, the interplay between action-sentence processing and motor processes (see related studies, reviewed in [132]). Together, three of the explored ERPs suggest that sentence and motor information are integrated in a bidirectional way.

## Supporting Information

Stimuli S1 Sentences lists.
(0.06 MB DOC)

Methods S1 Stimuli validation.
(0.03 MB DOC)

Methods S2 Selection of electrode position.
(0.02 MB DOC)

Table S1 N400-Like stimulus content × group interaction. Relevant comparisons are in bold. A significant effect of stimulus content was observed (F(2, 48) = 17.8; p<0.001). Post hoc analysis (MS = 1.207; df = 48) evidenced that NS were less negative (M = 0.014 µV, SD = 0.44) compared with OHS (M = −1.69 µV, SD = 0.61; p<0.001) and CHS (M = −1.38 µV, SD = 0.66; p<0.001). No difference between OHS and CHS was found (p = 0.58). However, an interaction effect between stimulus content × group was found (F(2, 48) = 48.31, p<0.001). Post hoc comparisons (MS = 1.21; df = 71.99) showed that the compatible stimuli in each group (OHS in the OHG and CHS in the CHG) were statistically different in terms of N400-like amplitudes. In brief, this last effect mirrored the category × group effect reported in the Results section.
(0.03 MB DOC)

Table S2 MP category × group interaction. Tukey HSD test, Approximate Probabilities for Post Hoc Tests Error: Pooled MS = 31.32, df = 70.42. Relevant comparisons are in bold. Post hoc comparisons performed over category × group interaction show enhanced compatibility effect in the CHG. Only CHG in the compatible condition was statistically different to the neutral and incompatible conditions, in terms of MP amplitudes.
(0.03 MB DOC)

Table S3 RAP: category × group interaction. Tukey HSD test; Approximate Probabilities for Post Hoc Tests Error: MS = 13.47, df = 70.54. Relevant comparisons are in bold. Post hoc comparisons performed over category × group interaction show that the compatible condition was statistically different from the neutral and incompatible conditions in CHG, in terms of RAP amplitudes. The OHG presented a trend towards significance in the incompatible condition compared with the compatible condition. The larger compatibility effect in CHG shown in MP was similar in RAP.
(0.03 MB DOC)
