# Supplementary material for: Applauding with Closed Hands: Neural Signature of Action-Sentence Compatibility Effects
Source: PLoS One. 2010 Jul 28;5(7):e11751. doi: 10.1371/journal.pone.0011751 (PMC2911376; doi:10.1371/journal.pone.0011751)
Supplement: Stimuli S1 — Sentences lists. (0.06 MB DOC) [file pone.0011751.s001.doc]

**Stimuli S1 (Sentences Lists)**

Note: English translations are only approximated. In the same line, the sentence structure as well as the target verb position, lexical properties, and idiomatic naturalness completely change in the translation. The Spanish sentences were all formed by a contextual phrase, a subject and a target past tense verb always as the last word.

List of neutral sentences. No manual action implied (NS).

1. Ese penal los llevaría a la final, Iván lo metió.

*The penalty would take them to the final, Ivan scored it.*

1. Su amigo le pidió prestado dinero, Pato se lo prestó.

*His friend asked him if he could borrow some money, so Pato loaned it to him.*

1. El viejo era sordo y no le escuchaba, Lucía le gritó.

*The old man was deaf and didn’t listen to her, so Lucía yelled at him.*

1. A su compañera le costaba el ramo, Inés le ayudó

*The signature was hard for her classmate, and Inés helped her.*

1. Entró un sospechoso al banco, el guardia lo vigiló.

*A suspect came into the bank, and the guard watched him.*

1. La película era tan triste que aunque estaba en el cine Fresia lloró.

*The movie was so sad that even though she was at the cinema, Fresia cried.*

1. La pintura era hermosa, por horas Agustín la contempló.

*The painting was beautiful; Agustín stared at it for hours.*

1. Se había quedado dormido en el sol, Fermín se insoló.

*He had fallen asleep in the sun, so Fermín got sunburned.*

1. Necesitaba el computador de su hermano, Laura se lo pidió.

*She needed her brother’s computer, so Laura asked him for it.*

1. Le cayó sopa caliente sobre las piernas, Lola se quemó.

*Hot soup was spilled all over her legs, and Lola was burned.*

1. No quería devolverse a su casa, Marieta se quedó.

*She didn’t want to get back home, so Marieta stayed.*

1. Su hermano la había traicionado, pero Aurora lo perdonó.

*Her brother had betrayed her, but Aurora forgave him.*

1. Debía comunicar la palabra del señor, en la plaza el evangélico predicó.

*He had to communicate the Word of the Lord, so the protestant preached at the Square.*

1. Debían resolver el caso del asesino, la policía lo investigó.

*They had to resolve the murderer’s case, so the police investigated.*

1. De los diez que habían solo él sabía hablar en inglés, Cristóbal les tradujo.

*Of the ten people only he could talk in English, so Cristobal translated for them.*

1. Se había demorado mucho en el baño, pero al fin Pepo terminó.

*He had taken too long in the toilet, but at last, Pepo went out.*

1. No creía en el fantasma que aparecía, pero esa tarde Eduardo lo vio.

*He didn’t believe in the ghost that kept appearing, but that evening Eduardo saw it.*

1. La micro paró justo adonde él iba, Danilo se bajó.

*The bus stopped just in front of the place he was going to, so Danilo got out.*

1. Nunca había probado la carne de soja, a Lulú le gustó.

*She had never tasted soya meat, but Lulu liked it.*

1. Le había hecho una pregunta que ya esperaba, Elena la respondió.

*He had asked him a question she was already waiting for, and Elena answered it.*

1. El niño se portó mal en el colegio, su padre lo castigó.

*The kid had bad behavior at school, so his father punished him.*

1. Necesitaba los 100.000 pesos que costaba su tele, Paloma la vendió.

*She needed the 100 dollars her TV cost, so Paloma sold it.*

1. Ya no usaba esos zapatos, Verónica los regaló.

*She did not use those shoes anymore, so Verónica gave them away.*

1. La difícil prueba sería al día siguiente, Jazmín estudió.

*The hard exam would be next day, so Jazmin studied.*

1. Todos estaban resfriados menos él, pero al final Jonás se contagió.

*Everyone caught a cold but him, but finally Jonas caught it.*

1. Nunca tomaba mucho alcohol, pero ese día Renato se emborrachó.

*He never drank too much alcohol, but that day Renato got drunk.*

1. Quería saber si la cantante se había separado, el periodista se lo preguntó.

*He wanted to know if the singer had divorced, so the journalist asked her.*

1. Su rostro era familiar, después de hacer memoria, Rafael se acordó.

*His face seems familiar to him, and after thinking, Raphael remembered him.*

1. El record era de 20 segundos, con 19 segundos Fernanda lo superó.

*The record was 20 seconds, but in 19 seconds, Fernanda broke it.*

1. Hace tiempo que quería ver a su abuela, Amaro la visitó.

*After waiting a long time to see his grandmother, Amaro visited her.*

1. El carcelero había dejado la reja abierta, el preso se escapó.

*The officer had left the cage open, and the prisoner escaped.*

1. Los niños le pidieron el cuento para dormir, Pía se los contó.

*The children asked her to read a bedtime story, so Pia told it to them.*

1. Su abuelo le mostró una pieza musical, Antonia la escuchó.

*Her grandfather showed her a musical piece, and Antonia listened to it.*

1. Se cayó en frente del niño que le gustaba, Pepita se avergonzó.

*She fell just in front of the kid she had a crush on, so Pepita was ashamed.*

1. Como tenía los cordones de las zapatillas sueltos, Hilda se cayó.

*Since she had her shoes untied, Hilda fell down.*

1. Los niños estaban gritando en la sala, la profesora los retó.

*The kids were screaming in the classroom, so the teacher scolded them.*

1. Su amiga quería leer ese libro, para el cumpleaños Amanda se lo regaló.

*Her friend wanted to read that book, so for her birthday Amanda gave it to her.*

1. No quería tomar una micro y aunque era lejos, Pipe caminó.

*He didn’t want to take the bus, so even though it was far, Pipe walked.*

1. El auto frenó ante el paso de cebra, el peatón cruzó.

*The car stopped before the zebra crossing, and the pedestrian crossed.*

1. Aunque le daba susto la montaña rusa, Toti se subió.

*Although she was afraid of the roller coaster, Toti got on it.*

1. El programa de computación era gratuito, Toño lo bajó.

*The software was free, so Toño downloaded it.*

1. El remedio era muy amargo, con dificultad Kika se lo tragó.

*The medicine was very bitter, but Kika swallowed it.*

1. Se sabía la canción que el amigo tocaba en la guitarra, Carla la cantó.

*She knew the song her friend was playing at the guitar, so Carla sang it.*

1. Tanto tiempo en la piscina le había dado frío, Braulio se salió.

*So much time in the pool had made him feel cold, so Braulio got out.*

1. Por mucho tiempo había buscado la fórmula, al fin el científico la encontró.

*He had searched for the formula for a long time, and at last, the scientist found it.*

1. Los médicos ya no podían hacer nada más, el enfermo se murió.

*There was nothing the doctors could do anymore, so the sick man died.*

1. Le tenía miedo a las arañas, apenas la vio en el suelo Aline la mató.

*She was afraid of spiders, but right after she saw one in the floor Aline killed it.*

1. No quería que sus ojos vieran esa escena, Jaime los cerró.

*He didn’t want his eyes to see that scene, so Jaime closed them.*

1. Tenía que llegar a la hora, pero había mucho tráfico, Juana se atrasó.

*She had to be there on time but the traffic was awful and Juana was late.*

1. En el florero habían unas flores muy aromáticas, Julia las olió.

*The flowers in the vase smelled good, so Julia smelled them.*

1. Necesitaba un cuaderno y ese era muy barato, Rosario se lo compró.

*She needed a notebook and found one that was very cheap, so Rosario bought it.*

1. El sofá se veía muy cómodo y quería descansar un poco, Paula se sentó.

*The sofa seemed very comfortable and she wanted to rest a little, so Paula sat on it.*

List of open hand-shape sentences (OHS)

1. El espectáculo era digno de alabanza, Rocío aplaudió.

*The show was praiseworthy, so Rocío applauded.*

1. Estaba aprendiendo a nadar de espalda, Daniela braceó.

*She was learning to swim the backstroke, so Daniela stroked.*

1. La bandeja estaba lista, el mozo la llevó.

Nombre:

*The tray was ready, so the waiter served it.*

1. El chaleco se le estaba llenando de migas, Juan se las sacudió.

*His sweater was getting full of crumbs, so John shook them off it.*

1. El tránsito estaba acelerado, el carabinero lo detuvo.

*The traffic was intense, but the police officer stopped it.*

1. Antes de tirar la pelota al aro, Ignacio la boteó.

*Before he threw the ball to the basket, Ignacio bounced it.*

1. Jugaban a las láminas, con un golpe Pedro las volteó.

*They were playing cards, and with a slap, Peter flipped them.*

1. El pelaje del gato era muy suave, Ángel lo acarició.

*The cat’s hair was very soft, and Angel stroked it.*

1. El auto se quedó en pana, Berta lo empujó.

*The car broke down, so Berta pushed it forward.*

1. El espejo estaba muy sucio, Tomás lo limpió.

*The mirror was very dirty, so Thomas cleaned it up.*

1. Con el paño debía dejar la mesa brillante, Mario lo pasó.

*He must leave the table shiny with the cloth, so Mario gave it a wipe.*

1. Se merecía esa cachetada, Pilar se la pegó.

*He deserved that slap, and Pilar slapped him.*

1. Quería tener el pelo con gomina, Javier se engominó.

*He wanted to have hair gel in his hair, so Javier gelled it.*

1. La iglesia estaba silenciosa para orar, Felipe rezó.

*The church was quiet enough to pray, so Phillip prayed.*

1. La gitana le iba a leer la mano, Josefa se la mostró.

*The gypsy was going to read her hand, so Josefa showed it to her.*

1. La caja estaba muy pesada, Valentina la cargó.

*The box was very heavy, but Valentina lifted it up.*

1. Tenía la cara con pintura, Lorena se la lavó.

*She got paint in her face, so Lorena washed it.*

1. De los tambores el bongó era su favorito, Andrés lo tocó.

*Of all drums, Bongo was his favorite, and Andrew played it.*

1. El perro no se merecía una palmada, pero Alejandra se la dio.

*The dog didn’t deserve a smack, but Alexandra gave it to him.*

1. La ceguera no le impedía saber cómo era su piel, Nicolás la palpó.

*Blindness didn’t prevent him from knowing what her skin was like, so Nicholas felt it.*

1. Sus ojos no podían ver algo tan feo, Violeta se los tapó.

*Her eyes couldn’t see such an ugly thing, so Violet covered them.*

1. Los contornos del jarrón estaban rugosos, el alfarero los alisó.

*The outlines of the vase were rough, and the potter smoothed them.*

1. El Mouse estaba trabado, pero Miguel lo arrastró.

*The Mouse was blocked, but Mike dragged it.*

1. La pelota de volley se elevó perfecta, Fernanda remachó.

*The Volleyball rose perfectly, and Fernanda spiked it.*

1. Para comenzar a hacer algo sobre la arena, Gabriel la emparejó.

*To begin doing something in the sand, Gabriel smoothed it.*

1. Le dolía la guata, Paco se la sobó.

*His belly hurt, so Paco rubbed it.*

1. Ya tenía la crema en las piernas, Catalina se la esparció.

*She already had the cream on her legs, so Catalina spread it.*

1. Hitler entró y con el debido gesto nazi el oficial lo saludó.

*Hitler came in, and with the appropriate Nazi gesture, the officer saluted him.*

1. La mosca se paró en la mesa, Verónica la aplastó.

*The fly stepped on the table, and Veronica smashed it.*

1. La guagua estaba llorando, su mamá la tomó.

*The baby was crying. His mother carried him.*

1. La ensaladera no cabía en la mesa, Oscar la sostuvo.

*The salad bowl didn’t fit on the table, so Oscar held it.*

1. Tenía muy acelerado el pecho, Víctor se lo presionó.

*His heart was racing, so Victor clutched his chest.*

1. El guante de baño tenía suficiente jabón, Hilda se jabonó.

*The bath towel had enough soap, so Hilda lathered herself.*

1. Con harto gel en la pierna, el entrenador se la friccionó.

*With a lot of gel on his leg, the trainer rubbed it down.*

1. Los números del ascensor estaban en braille, el ciego los leyó.

*The elevator’s numbers were on Braille, and the blind man read them.*

1. El polvo cubría el título del libro, Ariel se lo quitó.

*Dust covered the book’s title, so Ariel wiped it off.*

1. Quería elongar los brazos, Lucas los estiró.

*He wanted to stretch his arms, so Lucas stretched them.*

1. Pensó que el genio saldría de la lámpara, Aladino la frotó.

*He thought the Genie was getting out of the lamp, so Aladdin rubbed it.*

1. Quería saber si tocaba el techo, Laura se empinó

*She wanted to know if she could reach the ceiling, so Laura tiptoed.*

1. Inclinado hacia la meca, el musulmán oró.

*Facing toward Mecca, the Muslim prayed.*

1. No quería arena sobre su toalla prolijamente tendida, Juana se la sacó.

*She didn’t want sand over her tidily spread towel, so Juana took it off.*

1. Creía tener la tarjeta en el bolsillo, Inés la buscó.

*She thought she had the card in her pocket, so Ines searched for it.*

1. Solo la punta de sus dedos rozaba la moneda, Nora los extendió.

*Only her fingertips brushed the coin when Nora extended them.*

1. Quería ver si el anillo estaba debajo de la cama, Romina tanteó.

*She wanted to know if the ring was under the bed, so Romina felt for it.*

1. 20 flexiones de brazos serían suficientes, Eric las hizo.

*20 push-ups would be enough, Eric did them.*

1. La manicurista le pidió más espacio entre los dedos, Elena los abrió.

*The manicurist asked her for more space between the fingers, and Elena opened them.*

1. Quería parecer un pájaro volando, Fabián aleteó.

*He wanted to act like a flying bird, so Fabián flapped his wings.*

1. Jugaban a la pinta y uno se cayó, la que la llevaba lo pintó.

*They were playing “tag” and one of them fell down, and he was tagged.*

1. Jugando a la escondida llegó donde podía librarse, Marco se libró.

*Playing hide and seek, he reached the base and Marco freed himself.*

1. El cajón de gimnasia estaba muy alto, pero el gimnasta lo saltó.

*The Gym crate was very high, but the gymnast jumped over it.*

1. La plasticina aún tenía mucho relieve, Diego la aplanó.

*Plasticine has a lot of support, but Diego flatted it.*

1. Le regalaron un guante, Josefina se lo puso.

*They gave her a glove, and Josephine put it on.*

List of closed hand-shape sentences (CHS)

1. La vereda estaba llena de hojas secas

*The sidewalk was full of dry leaves, so John swept them.*

1. Se merecía un combo en la nariz

*He deserved a punch in the nose, and Joseph beat him.*

1. Con las manos asidas al volante en la esquina

*With hands gripping the steering wheel in the corner, Peter turned it.*

1. La mermelada se estaba pegando a la olla

*The jam was sticking to the pot, so the cook stirred it.*

1. La mugre de la camisa no salió con el lavado

*The muck on the shirt didn’t come out with washing, so Rodrigo brushed it.*

1. En el parque habían barras de fierro

*There were iron bars in the park, and Peter hung on them.*

1. En el partido de pin pon la pelota le venía alta

*In the ping-pong match, the ball was hit high in the air, and Nestor smashed it.*

1. Tenía los dientes sucios

*Her teeth were dirty, so Mary brushed them.*

1. Estaba junto al fierro cuando la micro frenó brusco

*She was next to the bar when the bus stopped suddenly, so Rosario held on to it.*

1. Había un cuchillo clavado en la madera

*There was a knife stuck into the wood, and Emma pulled it up.*

1. Tenía un cuchillo y una víctima

*He had a knife and a victim, and the murderer stabbed her.*

1. La manilla de la puerta estaba dura

*The door handle was locked, but Sergio turned it.*

1. La llave del agua estaba resbalosa

*The water valve was slippery, but Paz opened it.*

1. Los remos eran pesados

*The oars were heavy, but Anna rowed.*

1. Lo dejaba nocaut si le pegaba un gancho

*He would knock him out if he gave him a hook, so Martin beat him.*

1. La pala que le pidieron era muy pesada

*The shovel she was asked for was very heavy, but Andrea took it.*

1. Con la pluma fuertemente agarrada

*With the pen strongly seized, the poet wrote.*

1. Tenía que clavar el clavo muy derecho

*He needed to drive the nail correctly, so Joseph hammered it*

1. El animador no le quería pasar el micrófono

*The entertainer wouldn’t give him the microphone. The rocker took it from him.*

1. La opera es emotiva con binoculares

*The opera is emotive with binoculars, so Ada took them.*

1. La tapa de la botella estaba mala

*The screw top of the bottle was damaged, but Lucía turned it.*

1. El frasco de tempera seguía abierto

*The bottle of tempera was still open, so Bruno closed it.*

1. El cojín cabía dentro de su mano

*The cushion fit into his hand, and Clara clasped it.*

1. El mango de la espada brillaba

*The handle of the sword shined, and the Prince brandished it.*

1. El fierro estaba caliente

*The iron bar was hot, but Rosario grabbed it.*

1. Una mosca revoloteaba a su alrededor

*A fly was flying around her, but Magdalena caught it*

1. Los pelos de la brocha estaban duros

*The bristles of the paintbrush were stale, but Pepe painted.*

1. El mango del maletín era firme

*The handle of the briefcase was steady, so Beto lifted it.*

1. La barba ya estaba muy crecida

*His face was covered in hair, so Matías shaved himself.*

1. El ajo estaba en el mortero

*The garlic was on the mortar, and Viviana crushed it.*

1. El formulario estaba revisado

*The form was reviewed, and the secretary stamped it.*

1. El manubrio de la bici se movía mucho

*The handlebars of the bike were moving, and Sergio held on to them.*

1. El joystick era nuevo

*The joystick was new, so Bastian tried it.*

1. El tablero tenía una sola palanca

*The panel had only one gearshift, and Jaime triggered it.*

1. Temía pasar el cambio a tercera

*She was afraid to go to the third the gear, but Juana changed it.*

1. El quitasol se estaba saliendo de la arena

*The sunshade was coming off the sand, but Ximena buried it.*

1. Había que acumular la paja

*The straw had to be piled up, so Patricio raked it.*

1. La toalla estaba empapada

*The towel was soaped, and Macarena wringed it.*

1. Debía sacar jugo del pequeño limón

*She had to get juice out of the little lemon, so Rebeca squeezed it.*

1. El dibujo con grafito le quedó mal

*The graphite drawing was awful, so Memo rubbed it out.*

1. Tenía que embocar el palo en el agujero

*She had to hit the bar in the hole, and Natalia put it in the hole.*

1. Con la pala debía hacer un hoyo profundo

*With the shovel, he had to make a deep hole, so Abelardo sank.*

1. Con el dardo en la cerbatana y el blanco fijo

*With the dart in the peashooter and the target fixed, Antonio shot it.*

1. Las cuerdas de escalada no dejarían que cayera

*The rope wouldn’t let him fall down, so Danilo seized it.*

1. Ese coscacho estaba demás

*That punch was too much, but Paola gave it to him.*

1. Solo faltaba su firma en el contrato

*The only thing missing in the contract was his signature, so Christian signed it.*

1. Tenía el pelo desordenado

*Her hair was messy, so Violeta brushed it.*

1. Su pelo estaba muy enredado

*His hair was very tangled, so Omar combed it.*

1. Con la garrocha bien firme para saltar

*With the pole very steady to jump, the athlete drove himself.*

1. El papel arrugado se le cayo al suelo

*The crumpled paper fell on the floor, so Lucía picked it up.*

1. La ciruela ya había madurado en el árbol

*The plum had already ripened in the tree, and Manuela took it.*

1. Tras el salto de una barra paralela a la otra

*After the transition from one parallel to the other one, the gymnast held on.*
