# Supplementary material for: Applauding with Closed Hands: Neural Signature of Action-Sentence Compatibility Effects
Source: PLoS One. 2010 Jul 28;5(7):e11751. doi: 10.1371/journal.pone.0011751 (PMC2911376; doi:10.1371/journal.pone.0011751)
Supplement: Methods S1 — Stimuli validation. (0.03 MB DOC) [file pone.0011751.s002.doc]

**Methods S1 (Stimuli Validation)**

A) Relevant linguistic variables. To ensure that no unequal semantic processes (other than of the hand-shape content) caused response differences between lists, linguistics variables were controlled. All were third-person sentences whose critical verb was in *undefined preterit* tense (English’s simple past tense), and it was always placed as the last word of the sentence. Relevant linguistic variables were matched between lists (such as Transitivity, Situation Aspect, and content of clauses).

*Transitivity* was defined, following Hopper and Thompson [96], as a matter of scalar phenomenon instead of a clear-cut dichotomy. The Transitivity of the sentences in this study was classified in three balanced categories (*high Transitivity*, *medium Transitivity*, *low Transitivity*) ranked according to the ten semantic features of Transitivity proposed by the authors. The features are as follows: number of participants, kinesis, aspect, punctuality, volitionality, affirmation, mode, agency, affectiveness of the direct object and individuation of the direct object. A sentence is more transitive than another if it meets more of the features of the list (e.g. the sentence *se merecía un golpe en la nariz, José lo golpeó* / he *deserved a punch in the nose and Joseph beat him* has two participants -subject and direct object-, is telic (complete action), is highly kinetic, punctual (is non-durative) and volitional, is an affirmation, is indicative, the subject is high in potency, the object is totally affected and highly individuated. Therefore, this sentence is highly transitive.

Most of the sentences in the lists had high Transitivity. Only two sentences had low Transitivity and pertained to the NS list. A nonparametric Kruskal-Wallis test yielded no differences between sentence lists regarding Transitivity: H(2, 156)= 4.14; p= 0.12.

*Situation aspects* were defined as the meaning of the sentence as a type of situation expressed via the whole verbal context based on their aspect values. For the Situation Aspect classification of the sentences of the three lists, the taxonomy proposed by C. Smith [97] was used. Following this model, the types of Situation Aspects were balanced in this study and their parameters were:

*Activity*: dynamic, durative, atelic (e.g. *The show was praiseworthy, so Rocío applauded)*

*Accomplishment*: dynamic, durative, telic (e.g. *Con la pala debía hacer un hoyo profundo, Abelardo cavó/With the shovel, he had to make a deep hole, so Abelardo sank*)

*Achievement*: dynamic, non-durative, telic (e.g. *se merecía un golpe en la nariz, josé lo golpeó /* he *deserved a punch in the nose and Joseph beat him)*

*State*: static, durative (e.g. *No quería devolverse a su casa, Marieta se quedó /*

*She didn’t want to get back home, so Marieta stayed*). No states in action-sentences were expected because of their necessary dynamic propriety. Only two sentences were states and pertained to the NS list, whose neutrality permits the static parameter (NS was not necessarily action-sentences).

The *accomplishments* were the type of Situation Aspect present frequently in all of the lists. Only 2 sentences were *states* and pertain to NS the list. The other two types were balanced between lists. A nonparametric Kruskal-Wallis test yielded no differences between lists regarding Situation Aspect: H(2, 156)= 0.92, p= 0.63.

*Clauses*

For balance of the type of content and logic structure of the clauses, the following sentences were considered;

1) Sentences that had two verbs with an implicated hand-shape (e.g. *No quiso usar el timbre, Rosa tocó / she doesn’t want to ring the bell, Rose knocked the door*). (Sentences with this feature were excluded).

2) Sentences that had two or more animated subjects (e.g. *El niño podia tener fiebre, la madre lo tocó / The child could had a fever, his mother touched his forehead* – in this sentence *the child* and *his mother*). The few sentences with this feature were paired between lists (7 in OHS, 6 in CHS, 9 in NS).

3) Sentences that implied explicit causality (*le sangró la nariz porque Juan lo golpeó* / *he bled from his nose because John hit him*). (Sentences with this feature were excluded).

4) Sentences that implied an adversative (*La pala que le pidieron era muy pesada, pero Andrea la llevó/ The shovel she was asked for was very heavy, but Andrea took it*). The few sentences with this feature were paired between lists (5 in OHS, 7 in CHS, 6 in NS).

B) Frequency, Predictability, Prototypicality and Degree of manual specificity.

The frequency of use of final verbs was evaluated with the LIFCACH frequency software. All final target words presented moderate levels of frequency, except 5 sentences with moderate-low frequency, which were matched between lists (2 of OHS, 2 of CHS, 1 of NS).

The three lists were validated regarding the Predictability of the verb, and the two hand-shape lists were rated according to prototypicality and opening hand-shape degree, applying a questionnaire to 53 undergraduate students.

To determine whether context was predictive of final verb, subjects were asked to evaluate how fitting the final verb of the sentence was to the previous context using a 10-point Likert scale. Zero scores indicated that verbs were extremely unpredictable by their contexts and a score of 10 indicated high Predictability. To ensure the homogeneity of lists, sentences with low verb Predictability (under 95%) were eliminated (*M*= 8.67, *SD*= 0.09; *M*= 8.80, *SD*= 0.09, and *M=* 9.10, *SD*= 0.08; for OHS, CHS, and NS lists, respectively). An ANOVA yielded an effect of Predictability (F(2, 178)= 8.78, p< 0.01). Post hoc comparisons performed over this effect evidenced that NS have enhanced levels of Predictability compared with OHS (p< 0.001) and CHS (p< 0.001), but not between hand-shape sentences (p= 1.00). The hand-shape action lists did not differ in terms of verb Predictability (F(1, 51)= 1.94, p= 0.16).

As a measure of prototypicality of action hand-shape sentences, subjects were asked to evaluate, on a 1 (very easy) to 5 (very hard) rating scale, the difficulty of performance with the opposite hand-shape for the manual action encoded by the verb (e.g. to applaud with closed hands or to brush your teeth with open hands). All hand actions expressed in the sentences were highly prototypical of their shape because the two types of hand actions were difficult to perform with their opposite hand-shape. However, the CH actions (*M*= 4.46, *SD*= 0.9) seemed to be more difficult to perform with OH relative to OH actions with CH (*M*= 3.84, *SD*= 1.3). An ANOVA confirmed this difference (F(1, 51)= 86.39; p< 0.001) evidencing the higher prototypically of CS compared with OS.

To assure that participants interpreted the critical action as encoding the alleged hand-shape, a 10-point Likert scale (1-very closed to 10-very open) was applied to rate opening hand-shape degree. The lists presented accentuated ratings of open hand-shape degree for OH and accentuated degrees of closed hand for CH (F(1, 51)= 1945.66; p< 0.0001; CH (*M*= 2.47, *SD*= 0.10); OH (*M*= 8.40, *SD*= 0.11).
