# Supplementary material for: Applauding with Closed Hands: Neural Signature of Action-Sentence Compatibility Effects
Source: PLoS One. 2010 Jul 28;5(7):e11751. doi: 10.1371/journal.pone.0011751 (PMC2911376; doi:10.1371/journal.pone.0011751)
Supplement: Methods S2 — Selection of electrode position. (0.02 MB DOC) [file pone.0011751.s003.doc]

**Methods S2 (Selection of electrode position)**

To select the main site for ERP analysis, we performed a previously reported strategy for electrode selection [71,72]: A 3 (compatible, and neutral sentences) x 9 (electrode locations: Cz, C3, C4, Pz, P3, P4, Fz, F3 and F4) repeated-measures analysis of variance (ANOVA) around each ERP time window (N400, MP and RAP) was conducted.

*N400-like*. ANOVA yielded a significant ROI effect (F(8, 200)= 9.42, p< 0.05) and a significant category x location interaction, (F(16, 400)= 18.70, p< 0.01). Cz location presented the maximum negative amplitudes for the incompatible category (*M*= -3.64 µV, *SD*= 0.42).

*MP.* ANOVA yielded a significant ROI effect (F(8, 200)= 17.82, p< 0.01) and a significant category x location interaction (F(16, 400)= 21.67, p< 0.005). Cz location presented the maximum negative amplitudes for the compatible category (*M*= -28.37 µV, *SD*= 0.98).

*RAP.* ANOVA yielded a significant ROI effect (F(8, 200)= 14.68, p< 0.01) and a significant category x location interaction (F(16, 400)= 23.28, p< 0.005). Cz location presented the maximum positive amplitudes for the compatible category (*M*= 13.18 µV, *SD*= 0.82).

Not surprisingly, Cz was the canonical location for most of the N400 and Motor potential studies [66,73,74]. Consequently, we selected a ROI of 6 electrodes around Cz for further analysis.
