# Supplementary material for: Applauding with Closed Hands: Neural Signature of Action-Sentence Compatibility Effects
Source: PLoS One. 2010 Jul 28;5(7):e11751. doi: 10.1371/journal.pone.0011751 (PMC2911376; doi:10.1371/journal.pone.0011751)
Supplement: Table S1 — N400-Like stimulus content × group interaction. Relevant comparisons are in bold. A significant effect of stimulus content was observed (F(2, 48) = 17.8; p<0.001). Post hoc analysis (MS = 1.207; df = 48) evidenced that NS were less negative (M = 0.014 µV, SD = 0.44) compared with OHS (M = −1.69 µV, SD = 0.61; p<0.001) and CHS (M = −1.38 µV, SD = 0.66; p<0.001). No difference between OHS and CHS was found (p = 0.58). However, an interaction effect between stimulus content × group was found (F(2, 48) = 48.31, p<0.001). Post hoc comparisons (MS = 1.21; df = 71.99) showed that the compatible stimuli in each group (OHS in the OHG and CHS in the CHG) were statistically different in terms of N400-like amplitudes. In brief, this last effect mirrored the category × group effect reported in the Results section. (0.03 MB DOC) [file pone.0011751.s004.doc]

**Table S1 (N400-Like)**

| **Group** | **Sentence** | **{1}** | **{2}** | **{3}** | **{4}** | **{5}** | **{6}** |
| --- | --- | --- | --- | --- | --- | --- | --- |
| 1. OHG | CHS |  | **0,00** | **0,00** | 0,00 | 0,00 | 0,63 |
| 2. OHG | NS | **0,00** |  | 1,00 | 1,00 | 1,00 | 0,00 |
| 3. OHG | OHS | **0,00** | 1,00 |  | 1,00 | 1,00 | 0,00 |
| 4. CHG | CHS | 0,00 | 1,00 | 1,00 |  | 1,00 | **0,00** |
| 5. CHG | NS | 0,00 | 1,00 | 1,00 | 1,00 |  | **0,00** |
| 6. CHG | OHS | 0,63 | 0,00 | 0,00 | **0,00** | **0,00** |  |
