# Supplementary material for: Applauding with Closed Hands: Neural Signature of Action-Sentence Compatibility Effects
Source: PLoS One. 2010 Jul 28;5(7):e11751. doi: 10.1371/journal.pone.0011751 (PMC2911376; doi:10.1371/journal.pone.0011751)
Supplement: Table S2 — MP category × group interaction. Tukey HSD test, Approximate Probabilities for Post Hoc Tests Error: Pooled MS = 31.32, df = 70.42. Relevant comparisons are in bold. Post hoc comparisons performed over category × group interaction show enhanced compatibility effect in the CHG. Only CHG in the compatible condition was statistically different to the neutral and incompatible conditions, in terms of MP amplitudes. (0.03 MB DOC) [file pone.0011751.s005.doc]

**Table S2 (MP)**

| **Group** | **Category** | **{1}** | **{2}** | **{3}** | **{4}** | **{5}** | **{6}** |
| --- | --- | --- | --- | --- | --- | --- | --- |
| 1. OHG | Compatible |  | 0,16 | 0,14 | 0,03 | 0,01 | 0,02 |
| 2. OHG | Neutral | 0,16 |  | 1,00 | 0,00 | 0,90 | 0,93 |
| 3. OHG | Incompatible | 0,14 | 1,00 |  | 0,00 | 0,89 | 0,96 |
| 4. CHG | Compatible | 0,03 | 0,00 | 0,00 |  | **0,00** | **0,00** |
| 5. CHG | Neutral | 0,01 | 0,90 | 0,89 | **0,00** |  | 1,00 |
| 6. CHG | Incompatible | 0,02 | 0,93 | 0,96 | **0,00** | 1,00 |  |
