# Supplementary material for: Applauding with Closed Hands: Neural Signature of Action-Sentence Compatibility Effects
Source: PLoS One. 2010 Jul 28;5(7):e11751. doi: 10.1371/journal.pone.0011751 (PMC2911376; doi:10.1371/journal.pone.0011751)
Supplement: Table S3 — RAP: category × group interaction. Tukey HSD test; Approximate Probabilities for Post Hoc Tests Error: MS = 13.47, df = 70.54. Relevant comparisons are in bold. Post hoc comparisons performed over category × group interaction show that the compatible condition was statistically different from the neutral and incompatible conditions in CHG, in terms of RAP amplitudes. The OHG presented a trend towards significance in the incompatible condition compared with the compatible condition. The larger compatibility effect in CHG shown in MP was similar in RAP. (0.03 MB DOC) [file pone.0011751.s006.doc]

**Table S3 (RAP)**

|  | **Group** | **Category** | **{1}** | **{2}** | **{3}** | **{4}** | **{5}** | **{6}** |
| --- | --- | --- | --- | --- | --- | --- | --- | --- |
| **1** | OHG | Compatible |  | 0,13 | **0,06** | 0,02 | 0,00 | 0,00 |
| **2** | OHG | Neutral | 0,13 |  | 1,00 | 0,00 | 0,31 | 0,37 |
| **3** | OHG | Incompatible | **0,06** | 1,00 |  | 0,00 | 0,35 | 0,66 |
| **4** | CHG | Compatible | 0,02 | 0,00 | 0,00 |  | **0,00** | **0,00** |
| **5** | CHG | Neutral | 0,00 | 0,31 | 0,35 | **0,00** |  | 1,00 |
| **6** | CHG | Incompatible | 0,00 | 0,37 | 0,66 | **0,00** | 1,00 |  |
